# Evaluation of body composition monitoring for assessment of nutritional status in hemodialysis patients

**DOI:** 10.1080/0886022X.2019.1608241

**Published:** 2019-05-06

**Authors:** Haifen Zhang, Xingjuan Tao, Ling Shi, Na Jiang, Yan Yang

**Affiliations:** a Department of Nephrology, Renji Hospital, School of Medicine, Shanghai Jiao Tong University, Shanghai, China;; b School of Nursing, Shanghai Jiao Tong University, Shanghai, China

**Keywords:** Bioimpedance, body composition, lean tissue mass, nutrition assessment, end-stage renal disease

## Abstract

**Background:** Body composition monitoring is the only clinically available method for distinguishing among the three body components. This study aimed to determine the relationship between body composition and all-cause mortality in Chinese hemodialysis patients and examine whether the lean tissue index (LTI) derived from body composition monitoring can accurately diagnose malnourished patients.

**Methods:** Hemodialysis patients (*n* = 123) with nutritional and body composition assessment records in 2015 were examined. Body composition was assessed using a body composition monitor machine.

**Results:** Fifty-seven patients (46.3%) had low LTI (LTI less than the 10th percentile of the respective normal distribution). Significant differences in the fat tissue index (FTI) were observed, with the low LTI group having a higher FTI (10.8 kg/m^2^ vs. 9.0 kg/m^2^, *p*= .007). The kappa coefficient of agreement between LTI and subjective global assessment (SGA) was 0.26 for the presence of malnutrition. During the mean observation period of 26.7 months, 20 of 123 (16.3%) patients died. Low LTI remained highly predictive of survival in the Cox regression analysis (hazard ratio: 3.24, 95% confidence interval 1.06–9.91, *p*= .04). Malnourishment defined by SGA predicted survival in the Kaplan–Meier analysis (log-rank *χ*^2^=4.05; *p*= .04) but not in the multivariate analysis.

**Conclusions:** LTI is a predictor of mortality, and its predictive power was not affected when FTI, SGA, and hydration status were included in the multivariate analysis. However, SGA may not be adequate to identify patients at a risk of death among Chinese hemodialysis patients.

## Introduction

Malnutrition is highly prevalent in hemodialysis (HD) patients and results in impaired health-related quality of life, as well as increased risks of hospitalization, morbidity, and mortality [1–3]. Muscle mass wasting is the main manifestation of malnutrition for dialysis patients, it is therefore important to distinguish fat and muscle mass when performing nutritional assessment.

A body composition monitor (BCM) provides bedside assessment of volume status, lean tissue mass, and fat mass [4]. The values from the BCM on fluid management for HD patients have been extensively examined in both observational and experimental studies [5,6]. However, there has been little research on the appropriate use of BCM parameters for assessment of the nutritional status of HD patients. Several studies have documented that the lean tissue index (LTI) was a powerful predictor of mortality in HD patients [7–9]. Since the algorithms for cutoff points of LTI and fat tissue index (FTI) were derived from normal subjects and predominantly Caucasians, it remains unclear whether low LTI and/or FTI were associated with increased mortality in Asian HD patients. Recently, an observational study of 455 peritoneal dialysis (PD) patients revealed that even though BCM parameters (LTI and FTI) were significantly lower in malnourished PD patients being classified by subjective global assessment (SGA) than patients with normal nutrition, low LTI and/or FTI (below the 10th centiles of age, gender matched norm) were not able to diagnose patients with low SGA values [8]. Van Biesen et al. [10] observed that fat tissue mass was comparable between PD and HD, while muscle mass was better preserved for patients on PD as determined by BCM-derived LTI adjusting for diabetic status. Concerning differences in body composition distribution in patients on different dialysis modalities, whether observations of the relationship between LTI and SGA that were noted in PD patients are present in HD patients deserves further exploration.

This study aimed to explore the associations among BCM parameters, anthropometric measures, and SGA; and to determine the relationship between body composition and all-cause mortality in Chinese HD patients.

## Materials and methods

### Study design and participants

In this retrospective observational study conducted at the Dialysis Center of a tertiary care hospital in mainland China, all HD patients with a record of nutritional assessment and BCM measurement between September 2015 and December 2015 were potentially eligible for inclusion. The eligibility criteria of patients included being more than 18 years old and being on maintenance HD three times weekly for at least 3 months. Electronic medical records of the patients were retrieved anonymously in accordance with data protection rules after obtaining ethical approval from the ethic committee of the study hospital. Deaths occurring until June 2018 were analyzed for this study.

### Body composition

A BCM (Fresenius Medical Care, Sankt Wendel, Germany) was used to assess body composition. The measurement was carried out approximately 20–30 min before an HD session [7]. LTI, FTI, overhydration (OH), and body mass index (BMI) were retrieved from the BCM software. Normal LTI was defined as levels between the 10th and 90th percentile of the normal LTI distribution of the age and gender-matched healthy population. Low LTI was defined as under the 10th percentile of the respective normal distribution [9].

### Anthropometric measurements

Height and body weight were measured with the patient in light clothing. Triceps skin-fold thickness (TSF) was measured with a caliper on the non-access side of the patients. Mid-arm circumference (MAC) was measured by a tape at the midpoint between the acromion and the olecranon. Mid-arm muscle circumference (MAMC) was calculated based on MAC and TSF: MAMC (cm)=MAC (cm) – 3.14 × TSF (cm). Muscle strength was assessed by handgrip strength (HGS) with a hand-held dynamometer. Patients were instructed to complete the measures on the contralateral arm of the fistula arm in a standing position. All anthropometric measurements were taken about 10–15 min after the mid-week dialysis treatment by a trained nurse (SL).

### Subjective global assessment

SGA was determined based on the patient’s medical history and physical examination by the trained nurse (SL). Medical history included weight change, dietary intake change, functional impairment, gastrointestinal symptoms, and comorbidities. Evaluation of fat and muscle wasting, presence of edema, and ascites related to nutritional conditions were included in the physical examination. Patients were classified as grade A, well nourished, grade B, moderately malnourished, or grade C, severely malnourished. In this study, grade A was considered well-nourished, while grades B and C were grouped together as malnourished [11].

### Data analysis

The Statistical Package for the Social Science (SPSS) version 21.0 (SPSS Inc., Chicago, IL) was used for data analysis, and *p* values <.05 (two-tailed) were defined as statistically significant. Chi-square tests for categorical variables, independent sample *t*-tests, and Mann–Whitney’s *U* tests for continuous variables were adopted to compare the low LTI and normal LTI groups. Pearson’s or Spearman’s correlations were calculated to determine the correlation coefficients among LTI, FTI, and biochemical and anthropometric variables. Cohen’s kappa coefficient tests were used to determine the agreement between SGA and LTI for evaluating the presence of malnutrition. Malnutrition was defined as LTI value being under the 10th percentile of the respective normal distribution, or SGA being classified as grades B and C. A kappa value of 0.4 or less indicates low agreement, and a value of 0.7 or greater indicates high agreement [12]. The relationship among LTI, SGA, FTI, and serum albumin levels with all-cause mortality was analyzed using the Kaplan–Meier curves, log-rank test, and Cox proportional hazard model.

## Results

A total of 123 patients (56.9% males, median age of 64 years (interquartile range, IQR; 55–69)) were enrolled. Participant characteristics at baseline are summarized in Table 1. Fifty-seven patients (46.3%) had low LTI, and 87% of patients had normal FTI. Only six (4.9%) patients had both an LTI and FTI below the 10th centile of reference values. Low LTI and high FTI were found in 14.3% of male HD patients and 11.3% of females. The low LTI group had a higher FTI than the normal LTI group (*p* = .007). Patients in the low LTI group had lower BMI, MAMC, HGS, serum albumin, pre-albumin, and serum creatinine. A higher high-sensitivity C-reactive protein (CRP) level was observed in the low LTI group (Table 1).

**Table 1.** Patient characteristics and baseline information.

	Total	Low LTI (*n*= 57)	Normal LTI (*n*= 66)	*t*/Z/*χ*^2^	*p*
Age (years)	64.0 (55.0–69.0)	66.0 (58.0–72.5)	61.5 (53.0–68.0)	–1.632^b^	.10
Sex (*n*, %)					
Male	70 (56.9)	31 (25.2)	39 (31.7)	0.276^c^	.60
Female	53 (43.1)	26 (21.1)	27 (22.0)		
Dialysis vintage (months)	88.0 (46.0–143.0)	90.0 (48.5–143.0)	86.0 (44.0–141.0)	–0.540^b^	.59
Diabetes mellitus (*n*, %)					
Yes	25 (20.3)	12 (9.8)	13 (10.6)	0.035^c^	.85
No	98 (79.7)	45 (36.6)	53 (43.1)		
*Kt*/*V*	1.6 (1.5–1.8)	1.7 (1.6–1.9)	1.5 (1.5–1.7)	–2.566^b^	.01*
SCr (µmol/L)	1011.5 (163.8)	968.4 (158.5)	1048.7 (160.2)	2.783^a^	.006*
Albumin (g/L)	41.0 (3.3)	40.2 (3.6)	41.7 (2.8)	2.685^a^	.008*
Pre-albumin (mg/L)	331.3 (72.7)	311.7 (73.5)	348.0 (68.2)	2.866^a^	.005*
Hemoglobin (g/L)	110.4 (14.2)	110.0 (15.0)	110.8 (13.6)	0.302^a^	.76
Total cholesterol (mmol/L)	4.1 (1.0)	4.1 (1.0)	4.1 (1.1)	–0.134^a^	.89
Triglycerides (mmol/L)	2.4 (1.5–2.7)	2.4 (1.5–2.4)	2.4 (1.5–3.1)	–0.769^b^	.44
hs-CRP (mg/L)	2.5 (0.9–6.5)	3.7 (1.2–8.2)	2.1 (0.8–4.5)	–2.547^b^	.01*
SGA					
Malnutrition	69 (56.1)	40 (70.18)	29 (43.9)	8.548^c^	.003*
LTI (kg/m^2^)	12.5 (2.7)	10.6 (1.8)	14.1 (2.1)	9.902^a^	<.001*
FTI (kg/m^2^)	9.9 (3.7)	10.8 (3.9)	9.0 (3.3)	–2.746^a^	.007*
BMI (kg/m^2^)	22.8 (20.8–25.6)	21.9 (19.6–25.3)	23.4 (21.6–26.1)	–2.757^b^	.006*
WC (cm)	87.5 (10.1)	86.9 (11.3)	88.0 (9.1)	0.581^a^	.56
TSF (mm)	11.5 (8.5–14.0)	10.5 (8.5–13.5)	12.0 (8.5–15.0)	–1.153^b^	.25
MAMC (cm)	22.3 (20.3–23.6)	21.8 (20.1–22.9)	22.7 (20.7–24.1)	–2.721^b^	.007*
HGS (kg)	24.6 (10.3)	21.1 (10.4)	27.7 (9.3)	3.677^a^	<.001*

SCr: serum creatinine; hs-CRP: high-sensitivity C-reactive protein; SGA: subjective global assessment; LTI: lean tissue index; FTI: fat tissue index; BMI: body mass index; WC: waist circumference; TSF: triceps skinfold thickness; MAMC: mid-arm muscle circumference; Hb: hemoglobin; HGS: handgrip strength.

*Kt*/*V* represents the dose of hemodialysis, an abbreviation of (*K*_urea_×*T*_d_)/*V*_urea_. *K*_urea_ (mL/min).

^a^Independent *t*-test.

^b^Mann–Whitney’s *U*-test.

^c^*χ*^2^ test.

*p<.05.

LTI was positively correlated with HGS (*r*= 0.70, *p*< .001), MAMC (Spearman *ρ* = 0.47, *p* < .001), serum creatinine (Spearman *ρ* = 0.42, *p*< .001), serum albumin (*r*= 0.25, *p*= .005), and pre-albumin (*r*= 0.30, *p*= .001). Significant correlations were noted between FTI and BMI (Spearman *ρ* = 0.64, *p*< .001), TSF (Spearman *ρ* = 0.62, *p*< .001), and waist circumference (Spearman *ρ* = 0.70, *p*< .001). The correlations are presented in Table 2.

**Table 2.** Correlation coefficients of LTI and FTI with serum and anthropometric parameters.

Variables	LTI (kg/m^2^)	FTI (kg/m^2^)
BMI (kg/m^2^)	0.289**	0.644***
TSF (mm)	–0.066	0.622***
MAMC (cm)	0.469***	0.131
WC (cm)	0.048	0.695***
Albumin (g/L)	0.253**	–0.111
Pre-albumin (mg/L)	0.298**	–0.029
Triglycerides (mmol/L)	–0.069	0.234*
SCr (µmol/L)	0.417***	–0.054
HGS (kg)	0.699***	–0.340***

LTI: lean tissue index; FTI: fat tissue index; BMI: body mass index; TSF: triceps skinfold thickness; MAMC: mid-arm muscle circumference; WC: waist circumference; SCr: serum creatinine; HGS: handgrip strength.

*p< .05.

**p< .01.

***p< .001.

Patients categorized as malnourished by SGA showed significantly lower LTI (11.85 ± 2.55 vs. 13.24 ± 2.62; *p*= .004). There was a higher proportion of malnourished patients with an LTI below the 10th centile of the reference group (70.2% vs. 43.9%, *p*= .003). However, the agreement between LTI and SGA for the presence of malnutrition was poor with a kappa of 0.26. The results are presented in Table 3.

**Table 3.** Agreement between LTI and SGA regarding the presence of malnutrition.

	LTI^b^: positive	LTI: negative	All
	*n* (%)	*n* (%)	*n* (%)
SGA^a^: positive	40 (58.0%)	29 (42.0%)	69 (100%)
SGA: negative	17 (31.5%)	37 (68.5%)	54 (100%)

Correct classification rate: 62.6%, kappa 0.26.

^a^SGA: subjective global assessment.

^b^LTI: lean tissue index.

**Figure 1.**
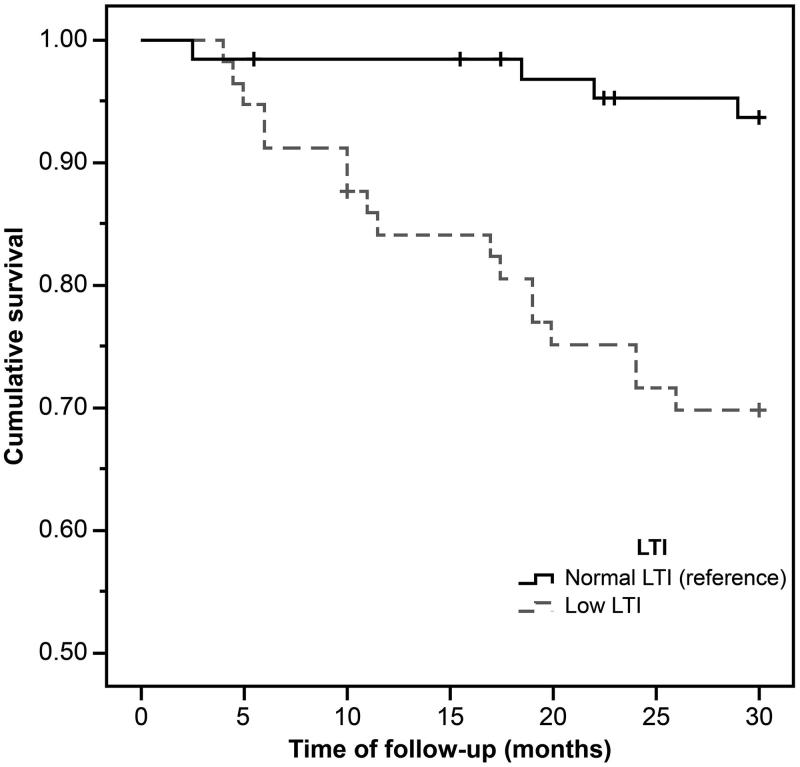
The Kaplan–Meier survival curves showing 30-month survival for patients in low and normal lean tissue index (LTI) groups. In patients in the low LTI group, mortality increased (*n* = 123; log-rank *χ*^2^=12.11; *p*=.001).

**Figure 2.**
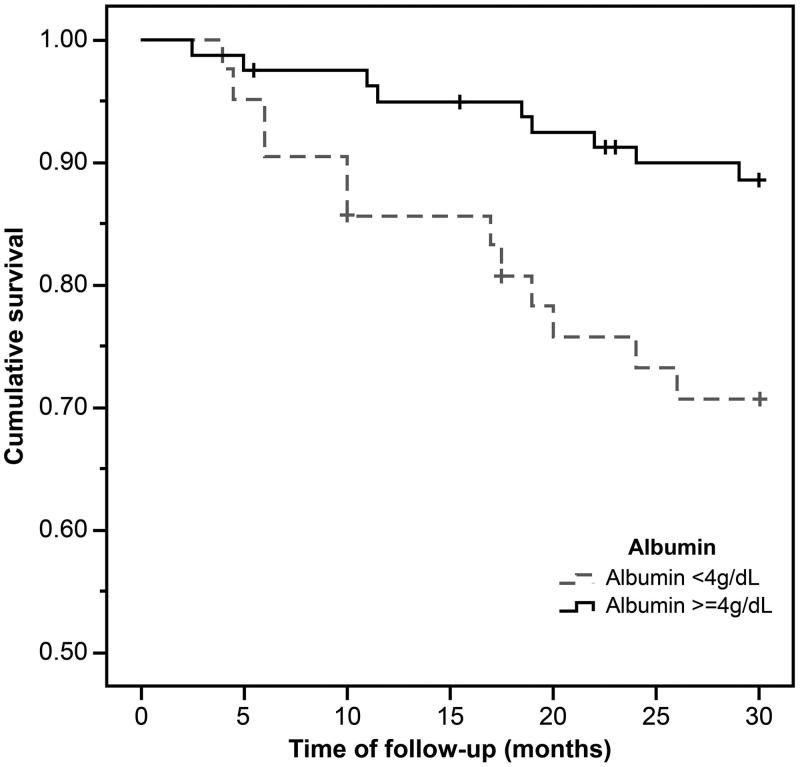
The Kaplan–Meier survival curves showing 30-month survival for patients in low and normal serum albumin group. In patients in the low serum albumin group, mortality increased (*n* = 123; log-rank *χ*^2^=6.42; *p*=.01).

**Figure 3.**
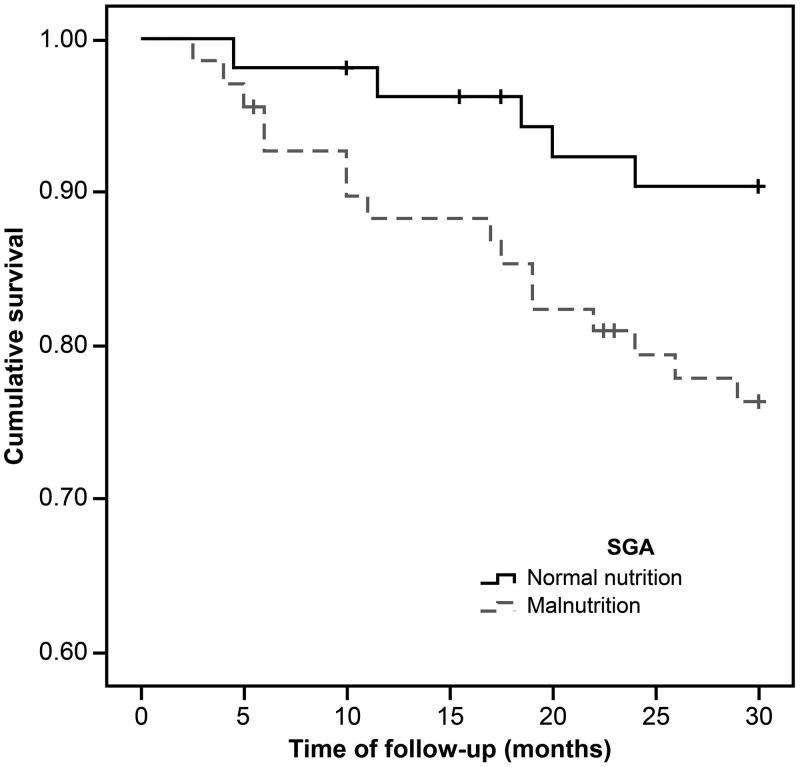
The Kaplan–Meier survival curves showing 30-month survival for patients diagnosed as normal and malnourished by SGA. In patients in the malnutrition group, mortality increased (*n* = 123; log-rank *χ*^2^=4.05; *p*=.04).

During the mean observation period of 26.7 ± 7.3 months, 20 out of 123 (16.3%) patients died, with 17 deaths (29.8%) from the low LTI group. Figures 1–3 show the Kaplan–Meier curves for different nutritional measures. Patients with low LTI (*p*= .001), serum albumin levels less than 4 g/dL (*p*= .01), and diagnosed as malnourished by SGA (*p*= .04) had a significantly higher risk of mortality. However, no significant survival differences were observed between the low and normal FTI groups. In the Cox regression analysis – adjusted for age, gender, dialysis vintage, and the presence of diabetes mellitus, serum albumin, OH, FTI, and SGA – low LTI remained significantly associated with all-cause mortality (hazard ratio: 3.24, 95% confidential interval: 1.06–9.91; Table 4); however, the relationship between SGA and mortality was not significant.

**Table 4.** Predictors of mortality derived from the Cox regression.

Risk factors	HR	95% CI	*p* Values
Age (years)	1.14	1.07–1.22	<.001
HD vintage (months)	1.02	1.00–1.02	.02
OH (L)	2.17	1.46–3.22	<.001
Low LTI (kg/m^2^)	3.24	1.06–9.91	.04

HR: hazard ratio; CI: confidence interval; HD: hemodialysis; OH: overhydration; LTI: lean tissue index; FTI: fat tissue index; SGA: subjective global assessment.

The Cox regression model is controlled for age, gender, dialysis vintage, presence of diabetes mellitus, OH, FTI, serum albumin, and SGA.

## Discussion

BCM is the only clinically available method for distinguishing among the three body components. The findings of this study support its value for early detection of alterations in muscle mass in HD patients as LTI was associated with multiple anthropometric and laboratory parameters. Even though muscle wasting is associated with malnutrition, and an LTI below the 10th centile of an age- and gender-matched reference group is a powerful predictor of mortality, the agreement between LTI and SGA for diagnosing malnutrition was poor.

As expected, low LTI group had significantly lower MAMC and HGS values in this study. Loss of muscle strength was accompanied by loss of muscle mass, and both muscle mass depletion and muscle weakness have been documented to be associated with reduced survival for dialysis patients [13,14]. On the other hand, Heimburger et al. reported a strong correlation between MAMC and lean body mass measured by dual X-ray absorptiometry [15]. MAMC is also included in the diagnostic criteria of protein-energy wasting as surrogates of muscle mass for chronic kidney disease [16]. The positive associations among LTI, MAMC, and HGS support the value of BCM for recognition of alterations in body composition in HD population. In all, 57 (46.3%) of the patients in this study had an LTI below the 10th centile of the reference values. The observations are in line with the findings reported by Marcelli et al. [9], in which half of the patients had an LTI below the 10th centile of the reference group. In the present study, only six (4.9%) patients had both an LTI and FTI below the 10th centile of reference values. Significant differences in FTI were observed, with the low LTI group presenting higher FTI than the normal LTI group. Low LTI and high FTI were found in 14.3% of male HD patients and 11.3% of females. These findings were in concordance with previous studies [9,17–19], and confirm that low muscle mass can occur despite fat mass accumulation, a phenomenon called ‘sarcopenic obesity’.

Although malnutrition is more frequently observed in the low LTI group, it may not be appropriate to use an LTI below the 10th centile of the distribution of reference values to diagnose malnutrition in Chinese HD patients. In our study, the kappa coefficient of the agreement between LTI and SGA was 0.26 for the presence of malnutrition, suggesting that the agreement between LTI and SGA was low. Similar observations were reported by a previous study, in which the sensitivity and specify of LTI to diagnose patients with low SGA was poor in 455 PD patients [8]. There are several possible reasons. First, it has been suggested that there may be two types of malnutrition in dialysis patients: malnutrition related to protein and energy intake and malnutrition associated with inflammation [20]. Chronic inflammation, as evidenced by increased inflammation markers such as CRP, has been shown to be associated with muscle wasting in HD patients [21]. Similar findings were observed in the current study. Only muscle mass was taken into consideration with the BCM-based malnutrition diagnosis, while an abnormal status of inadequate diet may not be captured. Second, patients who appear well-nourished according to anthropometric measures and subjective assessment can actually suffer from muscle wasting accompanied with fat mass accumulation. In our study, approximately 40% of patients with normal BMI had low LTI.

During the observation period, 20 of 123 (16.3%) patients died, with a mortality rate of 73.2 per 1000 patient-years. The overall mortality rate observed in this study was similar to that reported in previous studies [22,23], which was 76.8–82.0 per 1000 patient-years. In the adjusted model, patients with low LTI showed a significantly higher risk of mortality than patients with normal LTI. The findings are supported by a recent investigation in Poland [24], in which a worse one-year survival rate was observed in patients with low LTI in comparison with those with normal LTI. In line with the observations of the other researchers [7,25,26], older age, longer dialysis vintage, and OH were identified as significant predictors of mortality among dialysis patients in the present study. It is surprising that we failed to show an association between mortality and SGA in the multivariate Cox regression analysis after adjusting for variables such as age, gender, and the presence of DM. A possible explanation could be the gender-specific discrepancy that may exist in the association between SGA and mortality. Recently, Ko et al. [27] reported that a mortality risk of ‘mild-to-severe malnutrition’ evaluated by SGA was observed in male patients but not in female patients. In addition, results from a number of epidemiologic studies with large cohorts of HD patients indicated an ‘obesity paradox’, suggesting that obesity was associated with better survival [28]. In the present study, no significant association was found between FTI and mortality. In a sample of 37,345 HD patients, Marcelli et al. showed that patients with both LTI and FTI within the 10th–90th centiles of the healthy population had the best survival, and high FTI had protective advantages in patients with low LTI, whereas patients with low LTI and FTI had poorer outcomes [9]. It could be the ratio of lean to fat mass that exerts differential impact on health outcomes in the HD population.

The present study had certain limitations. First, it was a retrospective study with a relatively small sample size. Successive BCM measurements would be useful for determining the trend of body composition changes among HD patients and proposing corresponding nutritional advice. Second, measurement of BCM was performed before dialysis treatment. However, strong correlations were found between BCM parameters and anthropometric and biochemical measures, suggesting that BCM parameters measured before dialysis could be useful in health assessment in HD patients.

## Conclusions

BCM could be a valuable tool in the early detection of alterations in muscle mass. LTI is a powerful predictor of mortality, and its predictive power was not affected when FTI, SGA, and hydration status were included in the multivariate analysis. However, SGA may not be adequate to identify patients at a risk of death among Chinese HD patients.
